# Characteristics and clustering of human ribosomal protein genes

**DOI:** 10.1186/1471-2164-7-37

**Published:** 2006-02-28

**Authors:** Kyota Ishii, Takanori Washio, Tamayo Uechi, Maki Yoshihama, Naoya Kenmochi, Masaru Tomita

**Affiliations:** 1 Institute for Advanced Biosciences, Keio University, Tsuruoka, Yamagata 997-0035, Japan; 2 Graduate School of Media and Governance, Keio University, Fujisawa, Kanagawa 252-8520, Japan; 3 Graduate School of Information Science, Nara Institute of Science and Technology, Ikoma, Nara 630-0192, Japan; 4 Frontier Science Research Center, University of Miyazaki, Kiyotake, Miyazaki 889-1692, Japan; 5 Department of Environmental Information, Keio University, Fujisawa, Kanagawa 252-8520, Japan

## Abstract

**Background:**

The ribosome is a central player in the translation system, which in mammals consists of four RNA species and 79 ribosomal proteins (RPs). The control mechanisms of gene expression and the functions of RPs are believed to be identical. Most RP genes have common promoters and were therefore assumed to have a unified gene expression control mechanism.

**Results:**

We systematically analyzed the homogeneity and heterogeneity of RP genes on the basis of their expression profiles, promoter structures, encoded amino acid compositions, and codon compositions. The results revealed that (1) most RP genes are coordinately expressed at the mRNA level, with higher signals in the spleen, lymph node dissection (LND), and fetal brain. However, 17 genes, including the P protein genes (*RPLP0*, *RPLP1*, *RPLP2*), are expressed in a tissue-specific manner. (2) Most promoters have GC boxes and possible binding sites for nuclear respiratory factor 2, Yin and Yang 1, and/or activator protein 1. However, they do not have canonical TATA boxes. (3) Analysis of the amino acid composition of the encoded proteins indicated a high lysine and arginine content. (4) The major RP genes exhibit a characteristic synonymous codon composition with high rates of G or C in the third-codon position and a high content of AAG, CAG, ATC, GAG, CAC, and CTG.

**Conclusion:**

Eleven of the RP genes are still identified as being unique and did not exhibit at least some of the above characteristics, indicating that they may have unknown functions not present in other RP genes. Furthermore, we found sequences conserved between human and mouse genes around the transcription start sites and in the intronic regions. This study suggests certain overall trends and characteristic features of human RP genes.

## Background

The ribosome, which plays an important role in the translational mechanism, is universal to all organisms. Mammalian ribosomes consist of four RNA species and 79 ribosomal proteins (RPs) [1]. More than 2000 pseudogenes of RP genes are present in the human genome [2], and this has made it difficult to gain an overview of this gene family. However, we have already constructed a ribosomal protein gene database (RPG) [3,4] that contains the genomic DNA and full-length cDNA sequences. RPG also includes information on the transcription start sites, amino acid sequences encoded, and intron/exon structures, which has made it possible to conduct more systematic and detailed analyses of the RP genes from nine different eukaryotes.

In past studies, the control mechanisms of gene expression and RP functions were believed to be identical [5]. For example, most RP genes have common promoters [6] and were therefore assumed to have a unified control mechanism for gene expression [7]. Encoded amino acid and synonymous codon compositions [8] and G+C content [9] are also known to be similar in all RP genes. However, at this point it is unknown how many RP genes share typical features or which genes have specifically unique features.

In contrast, the protein structure and transcription mechanisms of individual RP genes have come to be gradually clarified through experimental investigation. In *Escherichia coli*, most RP genes are crucial for ribosome assembly, such as for the proteins implicated in the bridges between two subunits (RPS13, RPS15, RPS19, RPL2, RPL5, RPL14), contact with tRNA (RPS7, RPS9, RPS12, RPS13, RPL1, RPL5), and the surrounding polypeptide exit channel (RPL22, RPL24, RPL29) [10]. The presence of GC boxes [11] and binding sites for nuclear respiratory factor 2 (NRF-2) [12,13] and Yin and Yang 1 (YY1) [14] as transcription factor binding sites have been confirmed experimentally in the relevant RP genes in mammals. The binding site for activator protein 1 (AP-1) has been found in the downstream region of the transcription start site (TSS) of *Entamoeba histolytica RPL10 *[15]. A canonical TATA box is lacking near the TSS of the RP genes [13]. In addition, RPs have been found to have functions other than translation. It has been reported that *RPS3A *controls cell growth and apoptosis [16]. *RPL13A *controls translation silencing by itself [17]. Diverse RP gene expression control in specific tissues has also been reported using expressed sequence tag (EST) databases for humans [18] and catfish [19]. Investigation of the features of each RP gene has come to be one of the most important tasks in elucidating gene function, but few studies to date have used large-scale analysis to focus on the features of RP genes. We systematically analyzed the homogeneity and heterogeneity of RP genes on the basis of their expression profiles, promoter structures, encoded amino acid compositions, and codon compositions. We then attempted to extract the RP genes whose features differed from the set of typical features.

## Results

### Expression profile

**Figure 1:**
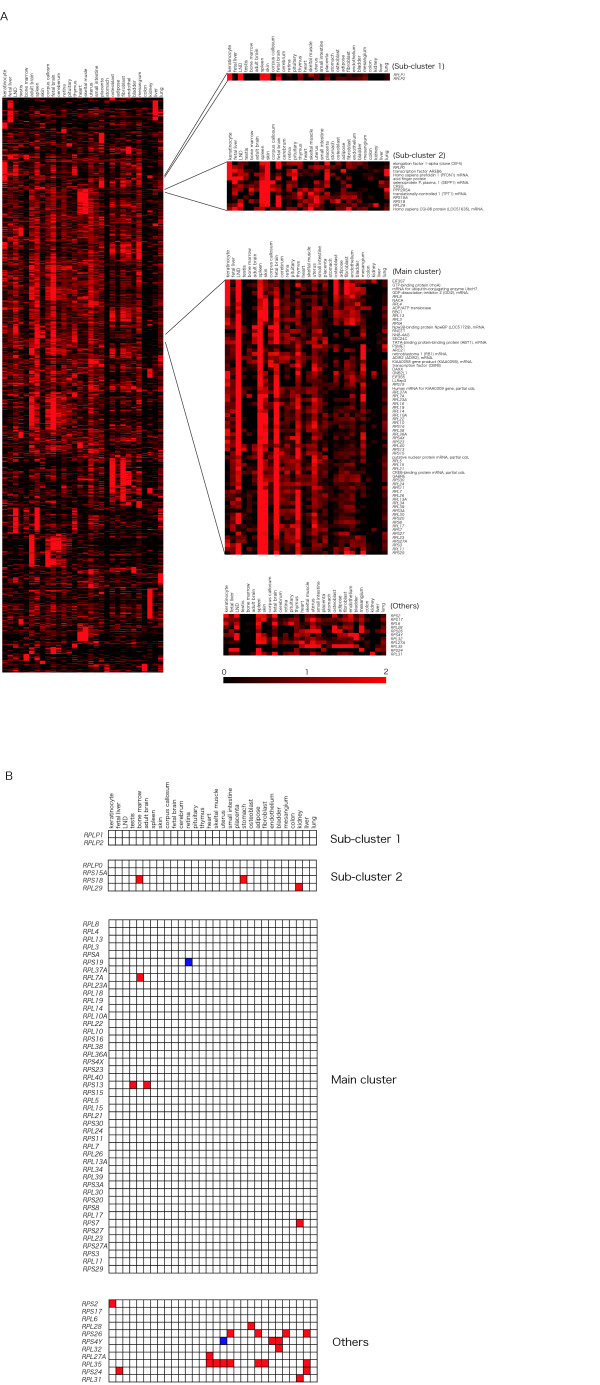
**Expression profiles of human ribosomal protein (RP) genes in 30 tissues**. (A) Hierarchical clustering of tissue expression profile using a centroid linkage algorithm. Black regions represent low gene expression level for that gene (row) in the tissue (column), whereas the red regions represent high gene expression level. The genes in the Main cluster, Sub-cluster 1, and Sub-cluster 2 show a correlation coefficient value of 0.94, 0.98 and 0.75, respectively.(B) Detection of differentially expressed RP genes by Grubbs' outlier test. Red regions represent genes (row) expressed at significantly higher rates in a certain tissue (column) than any other RP gene. Blue regions represent genes expressed at significantly lower rates than any other RP gene. A P value <0.05 was considered significant.

To investigate whether each RP gene expression pattern was identical, we performed cluster analysis with a large gene expression dataset (3281 genes, see Additional file 2). The RP gene expression patterns were classified into four classes; Main cluster, Sub-cluster 1, Sub-cluster 2 and the remaining 11 genes, which did not belong to any of these clusters (Fig. 1A), based on both the dendrogram generated by TreeView [20] and their expression patterns similarities. Original data files (CDT and GTR) to allow a reproduction of these clusters with dendrogram using the software TreeView have been made available (see Additional files 3 and 4). The Main cluster contained 46 RP genes, of which 28 encoded large subunit and 18 small subunit proteins, corresponding to 73% of the RP genes analyzed. These genes were relatively highly expressed in spleen, fetal brain, and LND. Furthermore, two translation initiation factor subunits (EIF3S5 and EIF3S7), both essential genes for translation machinery, were also present in the Main cluster (Table 1). Sub-cluster 1 consisted of *RPLP1 *and *RPLP2*, which were highly expressed in LND, keratinocytes, and skin. Sub-cluster 2 contained *RPS15A*, *RPS18*, *RPL29*, and *RPLP0*, which were expressed in skin, fetal brain, and spleen. Sub-cluster 2 was located nearer to Sub-cluster 1 than to the Main cluster. Eleven RP genes (*RPS2*, *RPS4Y*, *RPS17*, *RPS24*, *RPS26*, *RPL6*, *RPL27A*, *RPL28*, *RPL31*, *RPL32*, and *RPL35*) did not belong to any of these clusters. However, the expression patterns of *RPS2*, *RPS17*, and *RPL28 *were similar to that of the translation initiation factor EIF3S6, the translation elongation factor EEF1G, the putative translation initiation factor SUI1, and the ribosome associated membrane protein RAMP4. Furthermore, to investigate whether these 11 RP genes were expressed highly in different tissues than the other RP genes, we performed Grubbs' test using mRNA expression data (Fig. 1B). *RPL35 *was expressed more highly than the other RP genes in heart, skeletal muscle, uterus, small intestine, adipose tissue, fibroblasts, and liver. Nine of the 11 RP genes were highly expressed in tissues different from those showing the high levels of expression of the other RP genes. Although differentially expressed RP genes have been reported in humans [18], we demonstrated other RP genes with specific expression patterns. Bortoluzzi et al. (2001) analyzed expression profiles using the number of ESTs in UniGene [21]. On the other hand, our data was based on gene expression levels as measured by RT-PCR.

**Table 1:** Translation factors with similar expression patterns to those of RPgenes.

Genes related to translation	Cluster/RP Genes
EIF3S5	Main Cluster
EIF3S7	Main Cluster
EEF1A	Sub Cluster 2
EIF3S6	*RPS2*
EEF1G	*RPS17*
SUI1	*RPS17*
RAMP4	*RPL28*

### Prediction of transcription factor binding sites

**Figure 2:**
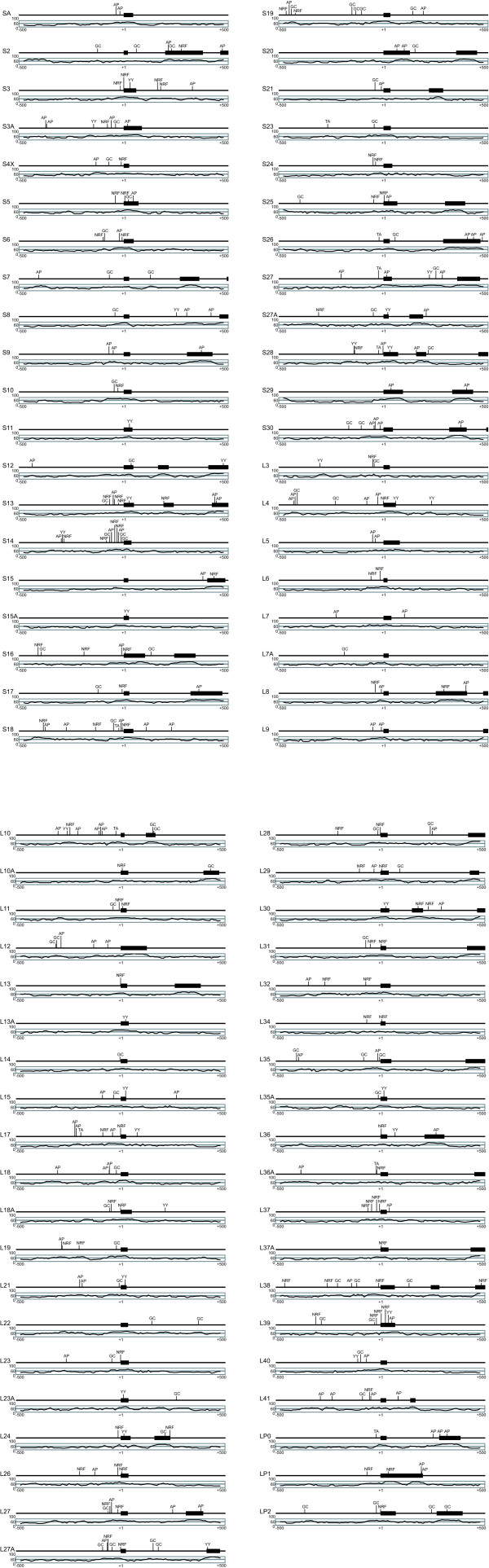
**Transcription factor binding sites predicted by phylogenetic footprinting**. The distance from the TSS (bp) is shown on the *X *axis, and the identity (%) with the orthologous mouse gene is shown on the *Y *axis. Black boxes indicate exons. Each thin line represents a location of a predicted promoter (NRF: nuclear respiratory factor 2; YY: Yin and Yang 1; GC: GC boxes; AP: activator protein 1; TA: TATA boxes).

**Figure 3:**
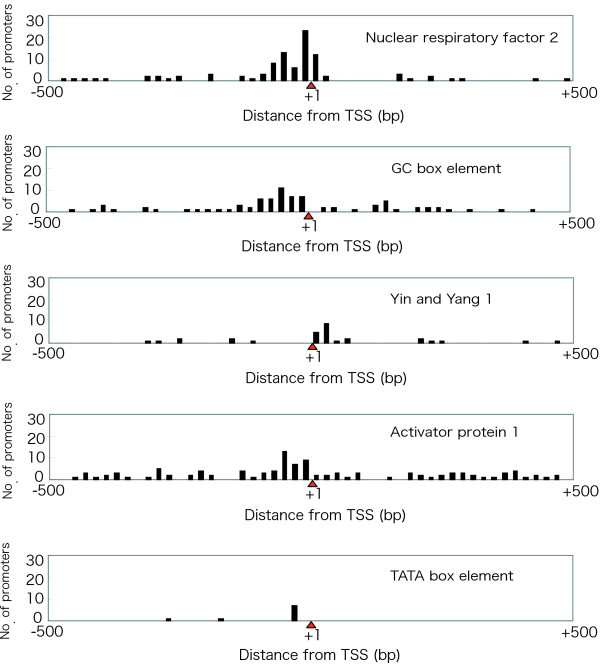
**Distribution of predicted transcription factor binding sites**. The distance from the TSS (bp) is shown on the *X *axis, and the number of predicted transcription factor binding sites is shown on the *Y *axis. The red triangle depicts TSS (+1).

We investigated the commonality and specificity of transcription initiation factors in the RP gene family by observing transcription factor binding sites (Fig. 2). Because our prediction was supported by phylogenetic footprinting between human and mouse, we expected that the candidates might possess higher reliability. Four promoters – NRF-2, GC box, YY1, and AP-1 – had already been demonstrated to have transcriptional activity in RP genes [6,11-15]. We found 95 binding sites for NRF-2 in 48 RP genes (Fig. 3). Most of the binding sites were located -80 bp to +20 bp from the TSS. Eighty GC boxes were found in 53 RP genes in upstream regions from -100 bp to -1 bp. Thirty binding sites for YY1 were found in 27 RP genes in downstream regions from +1 bp to +40 bp. There were 111 binding sites for AP-1 in 56 RP genes in upstream regions from -60 bp to -1 bp. On the other hand, only nine RP genes had TATA boxes, and seven (*RPS18*, *RPS26*, *RPS27*, *RPS28*, *RPL10*, *RPL36A*, and *RPLP0*) of these were predicted to have TATA boxes between -40 bp and -21 bp from the TSS in the upstream region. Nine RP genes had binding sites for all transcription factors. Twenty-nine RP genes had binding sites for three transcription factors, 22 had binding sites for two, and 19 had binding sites for one (Fig. 2). All RP genes were found to contain at least one transcription factor binding site. These data indicate that the common transcription factor binding sites in the RP genes were the GC box and the binding sites for NRF-2, YY1, and AP-1. In addition, we tried to find unknown transcription factor binding sites other than NRF-2, GC box, YY1, and AP-1 in the upstream regions of ribosomal protein genes. However, although a number were found, we did not consider them as actual sites, because we could not observe any specificity of these candidates for the RP genes.

### Amino acid composition

**Figure 4:**
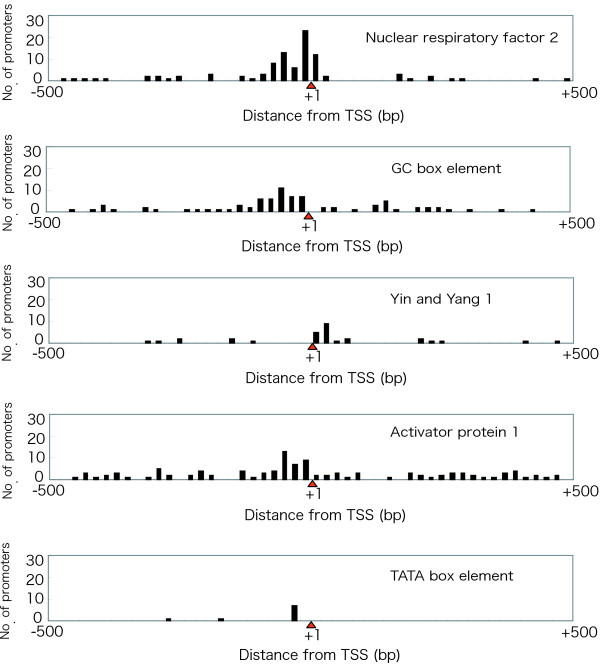
**Hierarchical clustering of ribosomal protein (RP) genes by amino acid composition**. The black regions represent low frequencies of encoding of the amino acid (column) by that gene (row) whereas the red regions represent high frequencies of encoding. (F: Phenylalanine, L: Leucine, I: Isoleucine, M: Methionine, V: Valine, P: Proline, T: Threonine, A: Alanine, Y: Tyrosine, H: Histidine, Q: Glutamine, N: Asparagine, K: Lysine, D: Aspartic acid, E: Glutamic acid, C: Cysteine, W: Tryptophan, R: Arginine, S: Serine, G: Glycine)

We analyzed the amino acids encoded by RP genes and classified the genes into groups by a clustering method. We performed cluster analysis using 80 human RP genes and 3000 genes selected randomly from RefSeq [21,22] (Fig. 4 and see also Additional files 6 and 7). The RP genes were divided into four classes: Main cluster, Sub-cluster 1, Sub-cluster 2, and others, based on both the dendrogram generated by TreeView and the similarities of amino acid composition. Sixty-two RP genes were present in the Main cluster. *RPLP1 *and *RPLP2 *were present in Sub-cluster 1 and *RPS29*, *RPL36A*, *RPL37*, *RPL37A*, and *RPL39 *were present in Sub-cluster 2. *RPSA*, *RPS3*, *RPS5*, *RPS12*, *RPS21*, *RPS26*, *RPS27*, *RPS28*, *RPLP0*, *RPL14*, and *RPL41 *did not belong to any of these clusters.

The average frequencies of lysine (0.13) and arginine (0.097) were highest of all the amino acids in the RPs. Lysine and arginine are basic amino acids. The frequencies of lysine and arginine in the Main cluster proteins were higher than those of the other 18 amino acids. The frequencies of lysine and arginine in the proteins encoded by *RPLP1 *and *RPLP2 *of Sub-cluster 1 were lower than their average frequencies in the proteins encoded by Main cluster genes.

The average frequencies of tryptophan (0.0077), cysteine (0.015), histidine (0.023) and methionine (0.026) were lowest of all the amino acids in RPs. Tryptophan, cysteine and methionine are neutral amino acids. This tendency was demonstrated more potently in proteins encoded by Sub-cluster 1 genes and less so in proteins encoded by Sub-cluster 2 genes.

### Synonymous codon composition

**Figure 5:**
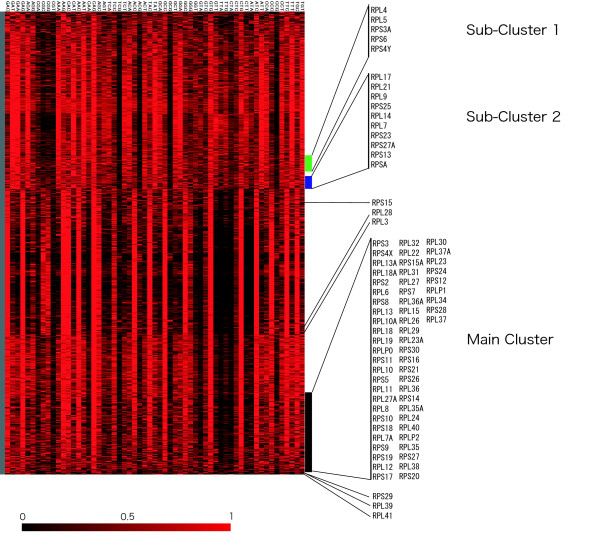
**Hierarchical clustering of synonymous codon composition**. The black regions represent low rates of occurrence of the codon (column) in that gene (row), whereas the red regions represent high rates of occurrence.

To evaluate which RP genes had come under similar selective pressure in the evolutionary process, we performed cluster analysis of the synonymous codon composition using the 80 human RP genes and 3000 genes randomly selected from RefSeq (Fig. 5 and see also Additional files 9 and 10). We found that the codon composition of the RP genes was divided into four classes (Main cluster, Sub-cluster 1, Sub-cluster 2, and Others), based on both the dendrogram generated by TreeView and the similarities of codon composition. Fifty-nine RP genes belonged to the Main cluster. In these RP genes the frequencies of AAG, CAG, ATC, GAG, CAC, and CTG were higher than those of any other codons. *RPS3A*, *RPS4Y*, *RPS6*, *RPL4*, and *RPL5 *were present in Sub-cluster 1. *RPS4X*, an isoform of *RPS4Y*, belong to the Main cluster, although they have similar amino acid composition.

In these RP genes the frequencies of GAT, GAA, CAG, AAG, GCT, ACT, and CAT were higher than those of any other codons. *RPSA*, *RPS13*,*RPL17*,*RPS23*,*RPS25*,*RPS27A*,*RPL7*,*RPL9*,*RPL14*, and *RPL21 *were present in Sub-cluster 2. The frequencies of AAG, CAG, TAT, GAA, GTT, ATT, GAT, AAT, CAC, and CTG were higher than those of any other codons.

AAG and CAG were frequently observed in all three clusters. The high frequency usage of these codons may be a common feature of RP genes. Codons with G or C in the third codon position were observed frequently in the Main cluster, distinguishing the Main cluster from Sub-cluster 1 and Sub-cluster 2. Furthermore, *RPS15*, *RPS29*, *RPL3*,*RPL28*, *RPL39*, and *RPL41 *did not belong to any cluster.

Forty-nine RP genes in the Main cluster on synonymous codon composition analysis also belonged to the Main cluster on amino acid composition analysis. Nine RP genes belonged to the Main cluster in terms of only the amino acids encoded. Seventeen RP genes belonged to the Main cluster in terms of only synonymous codon composition.

## Discussion

### BODYMAP expression profile data

The available human BODYMAP data obtained from the website [23,24] was already normalized in 30 human tissues. We downloaded all the data from the website and treated all of the data equally.

To evaluate the accuracy of our expression profile analysis, we made a comparison of BODYMAP expression profile data with mouse microarray data [25], downloaded from Gene Expression Omnibus (GEO) [26,27]. This mouse microarray data included 69 RP genes, and we observed one large RP gene cluster consisting of 39 genes in the expression profiles. We were also able to find a further 30 genes which did not belong to the cluster. Fifty-three of 69 RP genes in the mouse microarray data are included in the human BODYMAP data. In both datasets, 22 of 53 RP genes belong to the Main cluster and 10 of 53 RP genes did not. Therefore, the classification of more than 60 % (32 of 53 genes) of the genes with RP gene expression patterns was consistent between the two clustering analyses. Although the number of genes, species, type of tissues, and clustering method are different in the production of these two datasets, the classification of more than 60% of the RP genes was correspondence. Since the microarray data was measured by the ratio of the hybridization signal for each gene, it could vary by factors of 2 or greater. For such reasons, the expression level of each gene could not be compared. On the other hand, as the BODYMAP data was measured with PCR-based expression profiling method, it does indicate the relative concentration of gene transcripts in 30 human tissues. Therefore, tissue specific RP gene expression pattern can be determined by the BODYMAP data (Figure 1B). A similar bioinformatics approach the RP gene expression pattern has been performed by Bortoluzzi *et. al*. [18]. However, as their data was prepared from the public database UniGene, i.e., using assembled EST data recorded by many researchers, these data was not collected under the same conditions. They were able to observe specific RP gene expression patterns, but not RP genes with similar expression patterns. On the other hand, as our prepared BODYMAP data was measured under the same condition by one laboratory team (Okubo *et. al*.), we consider that BODYMAP data to be suitable for cluster analyses of the RP genes. For a better understanding of the BODYMAP data, we have provided the original data files (see Additional files 2, 3 and 4).

### Features of the major RP genes

From the results of our four analyses (expression profile, promoter prediction, encoded amino acids, and codon composition) we created a list of 80 human RP genes in rank order to form a "Feature Index" (FI) (Table 2). At least 24 RP genes with a FI of less than 1.0 in the list can be regarded as containing the features of the major RP genes. On the other hand, we consider RP genes with high FI scores to be specific RP genes.

**Table 2:** Feature indices of RP genes.

RP gene	Expression profile cluster	Amino acid encording cluster	Synonymous codon frequency cluster	TATA box (-40 bp ~ -21 bp)	Predicted Transcription factors bound	Feature Index
*RPLP0*	S2	O	M	Y	AP	3.3
*RPS26*	O	O	M	Y	GC, AP	3.2
*RPSA*	M	O	S2	N	AP	2.3
*RPS29*	M	S2	O	N	AP	2.3
*RPL5*	M	M	S1	N	AP	2.3
*RPL14*	M	O	S2	N	GC	2.3
*RPLP1*	S1	S1	M	N	NRF, AP	2.2
*RPLP2*	S1	S1	M	N	NRF, GC	2.2
*RPL36A*	M	S2	M	Y	NRF, AP	2.2
*RPS6*	-	M	S1	N	NRF, GC, AP	2.1
*RPS18*	S2	M	M	Y	NRF, GC, AP	2.1
*RPS27*	M	O	M	Y	GC, YY, AP	2.1
*RPL28*	O	M	O	N	NRF, GC, AP	2.1
*RPL41*	-	O	O	N	NRF, GC, AP	2.1
*RPS3A*	M	M	S1	N	NRF, GC, YY, AP	2.0
*RPS4Y*	O	M	S1	N	-	2.0
*RPS28*	-	O	M	Y	NRF, GC, YY, AP	2.0
*RPL4*	M	M	S1	N	NRF, GC, YY, AP	2.0
*RPL39*	M	S2	O	N	NRF, GC, YY, AP	2.0
*RPS15A*	S2	M	M	N	YY	1.3
*RPS23*	M	M	S2	N	GC	1.3
*RPS24*	O	M	M	N	NRF	1.3
*RPL6*	O	M	M	N	NRF	1.3
*RPL7*	M	M	S2	N	AP	1.3
*RPL9*	-	M	S2	N	AP	1.3
*RPL37A*	M	S2	M	N	NRF	1.3
*RPS15*	M	M	O	N	NRF, AP	1.2
*RPS21*	-	O	M	N	GC, AP	1.2
*RPL31*	O	M	M	N	NRF, GC	1.2
*RPL32*	O	M	M	N	NRF, AP	1.2
*RPL35*	O	M	M	N	GC, AP	1.2
*RPL37*	-	S2	M	N	NRF, AP	1.2
*RPS2*	O	M	M	N	NRF, GC, AP	1.1
*RPS3*	M	O	M	N	NRF, YY, AP	1.1
*RPS5*	-	O	M	N	NRF, GC, AP	1.1
*RPS12*	-	O	M	N	GC, YY, AP	1.1
*RPS17*	O	M	M	N	NRF, GC, AP	1.1
*RPS25*	-	M	S2	N	NRF, GC, AP	1.1
*RPL3*	M	M	O	N	NRF, GC, YY	1.1
*RPL17*	M	M	S2	N	NRF, YY, AP	1.1
*RPL21*	M	M	S2	N	GC, YY, AP	1.1
*RPL29*	S2	M	M	N	NRF, GC, AP	1.1
*RPS13*	M	M	S2	N	NRF, GC, YY, AP	1.0
*RPS27A*	M	M	S2	N	NRF, GC, YY, AP	1.0
*RPL10*	-	M	M	Y	NRF, GC, YY, AP	1.0
*RPL27A*	O	M	M	N	NRF, GC, YY, AP	1.0
*RPS9*	-	M	M	N	AP	0.3
*RPS11*	M	M	M	N	YY	0.3
*RPL7A*	M	M	M	N	GC	0.3
*RPL13*	M	M	M	N	NRF	0.3
*RPL13A*	M	M	M	N	YY	0.3
*RPL22*	M	M	M	N	GC	0.3
*RPL34*	-	M	M	N	NRF	0.3
*RPS7*	M	M	M	N	GC, AP	0.2
*RPS10*	-	M	M	N	NRF, GC	0.2
*RPS20*	M	M	M	N	GC, AP	0.2
*RPS30*	M	M	M	N	GC, AP	0.2
*RPL8*	M	M	M	N	NRF, AP	0.2
*RPL10A*	M	M	M	N	NRF, GC	0.2
*RPL11*	M	M	M	N	NRF, GC	0.2
*RPL12*	-	M	M	N	GC, AP	0.2
*RPL18*	M	M	M	N	GC, AP	0.2
*RPL23A*	M	M	M	N	GC, YY	0.2
*RPL26*	M	M	M	N	NRF, AP	0.2
*RPL35A*	-	M	M	N	GC, AP	0.2
*RPS4X*	M	M	M	N	NRF, GC, AP	0.1
*RPS8*	M	M	M	N	GC, YY, AP	0.1
*RPS16*	M	M	M	N	NRF, GC, AP	0.1
*RPS19*	M	M	M	N	NRF, GC, AP	0.1
*RPL15*	M	M	M	N	GC, YY, AP	0.1
*RPL18A*	-	M	M	N	NRF, YY, GC	0.1
*RPL19*	M	M	M	N	NRF, GC, AP	0.1
*RPL23*	M	M	M	N	NRF, GC, AP	0.1
*RPL24*	M	M	M	N	NRF, YY, GC	0.1
*RPL27*	-	M	M	N	NRF, GC, AP	0.1
*RPL30*	M	M	M	N	NRF, YY, AP	0.1
*RPL36*	-	M	M	N	NRF, YY, AP	0.1
*RPL38*	M	M	M	N	NRF, GC, AP	0.1
*RPL40*	M	M	M	N	GC, YY, AP	0.1
*RPS14*	-	M	M	N	NRF, GC, YY, AP	0.0

The feature index (FI) is a quantitative measure of the heterogeneity in an individual RP gene. Expression profile, amino acids encoded, and synonymous codon composition: a value of 1 was given to genes that did not belong to the Main cluster. TATA box: a value of 1 was given to genes that had TATA boxes. Common promoter: the maximum value was set as 0.4, because no obvious clusters were found for the analysis of promoter prediction. Then, if a binding site for one of four common transcription factors (nuclear respiratory factor 2 (NRF), GC boxes (GC), Yin and Yang 1 (YY), and activator protein 1 (AP)) was found, a value of 0.1 was subtracted. The columns "Expression profile cluster", "Amino acid encoding cluster", and "Synonymous codon frequency cluster" indicate the clusters to which RP genes were assigned as a result of each analysis. M: Main cluster; S1: Sub-cluster 1; S2: Sub-cluster 2; O: Other. The column "TATA box" indicates the existence of a TATA box, Y: Yes, N: No. For example, FI 3.3 of *RPLP0 *was calculated as follows; +1.0 (Expression profile), +1.0 (Amino acid encoding cluster), +0 (Synonymous codon frequency), +1.0 (TATA box), +0.4 -0.1 (Predicted Transcription Factors bound).

The features of the major RP genes gradually became clear to us from the four analyses. We were thus able to make the following four points in relation to typical features. (1) In the spleen, LND, and fetal brain the major RP genes are highly expressed; the control mechanism of regulation in these tissues might be different at the post-transcription level as reported in previous study [19]. (2) Major RP genes have GC boxes and possible binding sites for NRF-2, YY1, and/or AP-1. However, they do not have canonical TATA boxes. The AP-1 transcription factor is mainly composed of Jun, Fos and ATF protein dimers, which are thought to regulate the processes of proliferation, differentiation, apoptosis and transformation [28,29]. Their activity was confirmed in *Entamoeba histolytica RPL10 *[15] and their homologues were confirmed in mammals. Moreover, since their consensus sequence of the human AP-1 binding site (CGTGAGTCATG) was similar to that of *Entamoeba histolytica RPL10 *[15], the existence of the AP-1 transcription factor binding sites can also be putatively accepted in human RP genes. Though analyzed in detail, we observed no clear relationship between the results of the expression profile analysis and promoter prediction. (3) The major RP genes show a characteristic encoded amino acid composition of high lysine and arginine content. RPs, which interact with rRNA in the ribosome complex, has been suggested to have many arginines and lysines on the surface. (4) Major RP genes show a characteristic synonymous codon composition with a high rate of G or C in the third codon position and a high content of AAG, CAG, ATC, GAG, CAC, and CTG. It is believed that the species and number of tRNAs in the genome influence the compositional bias for codon selection [30].

Although the features noted here for the major RP genes were what had already been believed in general, these results confirm the major features of the RP genes within a whole set. Moreover, our results have revealed that RP genes that do not belong to the major groups do exist among the 80 RP genes; the unique features of these genes should prove useful to the field for the course of further study.

### Features of specific RP genes

At least 12 RP genes with a FI score of greater than 2.1 can be regarded as specific RP genes. Their unique features are listed in table 3 and discussed in detail in the following sections.

**Table 3:** Features of specific RP genes.

RP genes	Features
*RPLP0*	Unique gene expression profile was observed. Amino acid composition was unique.
*RPLP1, RPLP2*	They were highly expressed in LND and keratinocytes. The frequencies of lysine and arginine were low.
*RPL41*	The size was shortest. Amino acid composition was unique. The level of GC_3 _was lowest, and codon composition was unique.
*RPSA*	The frequencies of lysine and arginine were low. Codon composition was unique.
*RPS6*	Codon composition was unique.
*RPS18*	It was highly expressed in bone marrow and stomach.
*RPS26*	It was highly expressed in small intestine, adipose mesangium and liver. The frequency of lysine was low.
*RPS27*	The frequencies of lysine and arginine were low.
*RPS29*	The frequency of lysine was low. Codon composition was unique.
*RPL5*	Codon composition was unique.
*RPL14*	The frequency of arginine was low. Trinucleotide (GCT) repeats was contained.
*RPL28*	It was highly expressed in osteoblast. Codon composition was unique.
*RPL36A*	The frequency of cysteine was high.

The feature index (FI) is a quantitative measure of the heterogeneity in an individual RP gene. Expression profile, amino acids encoded, and synonymous codon composition: a value of 1 was given to genes that did not belong to the Main cluster. TATA box: a value of 1 was given to genes that had TATA boxes. Common promoter: the maximum value was set as 0.4, because no obvious clusters were found for the analysis of promoter prediction. Then, if a binding site for one of four common transcription factors (nuclear respiratory factor 2 (NRF), GC boxes (GC), Yin and Yang 1 (YY), and activator protein 1 (AP)) was found, a value of 0.1 was subtracted. The columns "Expression profile cluster", "Amino acid encoding cluster", and "Synonymous codon frequency cluster" indicate the clusters to which RP genes were assigned as a result of each analysis. M: Main cluster; S1: Sub-cluster 1; S2: Sub-cluster 2; O: Other. The column "TATA box" indicates the existence of a TATA box, Y: Yes, N: No. For example, FI 3.3 of *RPLP0 *was calculated as follows; +1.0 (Expression profile), +1.0 (Amino acid encoding cluster), +0 (Synonymous codon frequency), +1.0 (TATA box), +0.4 -0.1 (Predicted Transcription Factors bound).

### RPLP0, RPLP1, RPLP2

Animals, insect, fungi and protozoans possess three classes of acidic ribosomal P proteins: RPLP0, RPLP1 and RPLP2 [31-33]. It is reported that the heterodimers of RPLP1α/RPLP2β and RPLP1β/RPLP2α form stalk in the 60S large subunit with RPLP0 in the yeast [34]. On the other hand, the heterodimer of RPLP1 and RPLP2 form stalk in the silkworm [32]. P protein complex binds to the GTPase domain of rat 28 S rRNA in a buffer containing Mg^2+ ^[35]. It is also known that phosphorylated P proteins interact with elongation factor EF-2 in the rat [36,37].

Interestingly, *RPLP1 *and *RPLP2 *have their own specific characteristics on both expression profiling and amino acid composition by our analyses. In our expression profile, *RPLP1 *and *RPLP2 *were highly co-expressed in LND and keratinocytes, forming a sub-cluster. As only RPLP1 and RPLP2 form dimers in the silkworm, they may have gene expression machinery different from those of the other RP genes. In addition, they also belonged to the same sub-cluster in the study of encoded amino acid composition. In this cluster, the average frequencies of encoded lysine and arginine were lower than for the main RP genes, indicating a possible cause for the RPLP1 and RPLP2 location "stalk" in the ribosome complex. Although the P protein conformation is constructed from three proteins, interestingly, *RPLP0 *did not belong to the Main cluster or Sub-cluster 1 (which contained only *RPLP1 *and *RPLP2*) in either the expression profile or amino acid composition studies. *RPLP0 *was predicted to have a TATA box in the upstream region of TSS. Therefore, this may indicate that *RPLP0 *is a specific gene not only for P proteins but also for the RP gene family. On the other hand, because all three P protein genes belonged to the Main cluster in the study of synonymous codon composition, evolutionarily they might have been affected by selective pressure on codon usage along with other RP genes. From these results, we conclude that *RPLP0*, *RPLP1*, and *RPLP2 *are unique and specific genes compared with the major RP genes, but that these P protein genes are members of the RP gene family.

### RPL41

*RPL41 *was one of the RP genes with higher specificity (FI = 2.1). The coding sequence (CDS) size of human *RPL41 *was shortest (78 bp) among all the RP genes, the average size being 521 bp. Human *RPL41 *was independent from the Main cluster in terms of the encoded amino acid composition (Fig. 4) and synonymous codon composition (Fig. 5), although we applied codon usage data less affected by amino acid composition [38]. On the specificities of synonymous codon composition, we calculated the GC_3 _level (the frequency of G or C in the third codon position) in light of the suggestion that the short length of *RPL41 *could have biased the synonymous codon composition. The average GC_3 _level in human RP genes was 53.1%. In contrast, the GC_3 _level of *RPL41 *was 23.1%, the lowest of all the RP genes. Therefore, it is likely that the specificities of synonymous codon composition was scarcely affected by biased amino acid composition, or by the shortness of *RPL41*, but rather, was solely affected by differential evolutionary pressure unlike the other RP genes. Removal of yeast RPL41 did not affect the ratio of 60 S to 40 S subunits, but it did reduce the amount of 80 S, suggesting that RPL41 was involved in ribosomal subunit association [39]. As *RPL41 *is known to be dispensable in yeast [39], we consider it possible that human *RPL41 *also helps solely in association with ribosomal subunits. Although human *RPL41 *is known as one of the RP genes, our data indicates that it may not be a typical RP gene.

### Other specific RP genes

The FIs of *RPSA*, *RPS6*, *RPS18*, *RPS26*, *RPS27*, *RPS29, RPL5*, *RPL14*, *RPL28*, and *RPL36A *were higher than those of the other RP genes. Some of these RP genes had specificity in terms of the amino acids encoded, with lower frequencies of encoded lysine (*RPS26*, *RPS29*), arginine (*RPL14*), or both (*RPSA*, *RPS27*). In addition, *RPL14 *contains an array of 10 repeats of the trinucleotide GCT that encodes a polyalanine tract in the 3'-flanking sequence. As this polyalanine is conserved only in humans and mice, this characteristic sequence would seem to have been inserted in *RPL14 *during the evolution of these species. *RPS26 *did not belong to any cluster in either the expression profile or the encoded amino acid composition study. Moreover, it was predicted not to have the four typical promoters, but to contain the TATA box. Interestingly, it was found to belong to the Main cluster in the study of synonymous codon composition, indicating that *RPS26*, like the other RP genes, was affected by selective pressure on codon usage during the course of evolution. Consequently, these specificities suggest that these RP genes may have functions in addition to translation of which we are not yet aware.

### Conserved regions in mouse RP genes

Conserved regions with lengths of over 100 bp were found in regions upstream of the TSS in the following RP genes: *RPS2*, *RPS4X*, *RPS7*, *RPS10*, *RPS12*, *RPS14*, *RPS18*,*RPS23*, *RPS27A*, *RPS30*, *RPL6*, *RPL7*, *RPL10*, *RPL15*, *RPL17*, *RPL18*, *RPL19*, *RPL21*, *RPL22*, *RPL26*, *RPL27A*, *RPL32*, *RPL35*, *RPL35A*, *RPL36A*, *RPL40*, and *RPLP1*. Most importantly, 14 RP genes were found to have conserved upstream regions of over 100 bp adjacent to the TSS. Conserved intronic regions with lengths of over 100 bp were found in *RPS3*,*RPS6*,*RPS8*,*RPS19*,*RPS27*,*RPL7*,*RPL22*,*RPL23A*, and *RPL30*. Moreover, there were no transcription factor binding sites in *RPS6 *and *RPL23A*, suggesting that these intronic regions were conserved because of the existence of the following characteristics: (1) specific regulatory elements; (2) small nucleolar RNAs (snoRNAs), a type of non-coding RNA; (3) repetitive elements such as transposons; and (4) unidentified alternative exons. We confirmed that the conserved intronic region in *RPS8 *contains snoRNA, which functions in Box C/D 2'-*O*-methylation, from +289 bp to +368 bp [9]. For this reason, these conserved regions are likely to have certain biological functions.

### Synonymous codon bias in RP genes

In *E. coli*, *Schizosaccharomyces pombe*, and *Caenorhabditis elegans*, the synonymous codon is highly biased according to the tRNA-gene copy numbers [30]. On the other hand, in *Drosophila melanogaster *and *Homo sapiens*, codon composition is influenced largely by the number of GC-dinucleotides, rather than by the selective pressure on codon usage attributable to the number of tRNAs [30]. Furthermore, in higher vertebrates such as humans, a major factor contributing to codon usage is the variation in the long-range GC level, the isochore [30]. We conducted principal component analysis only for the RP genes in *E. coli*, *Methanococcus jannaschii*, *Saccharomyces cerevisiae*,*C. elegans, D. melanogaster*, and *H. sapiens *with codon usage data, called relative adaptiveness (W) [40]. The results indicated homogeneity of codon composition in the RP genes of *E. coli*, *M. jannaschii*, *S. cerevisiae*, and *C. elegans *(see Additional file 1). Therefore, most of the RP genes in these species were affected by translational selection. On the other hand, heterogeneity of codon composition was observed in the RP genes of *D. melanogaster *and *H. sapiens *[30]. These results are also consistent with the results of our cluster analysis of codon composition; many RP genes (26%) did not belong to the Main cluster (Fig. 5). These results imply that the number of RP genes affected by different selective pressures increased gradually during the evolutionary process from prokaryote to human. Because higher eukaryotes may have gained several factors such as the isochore, the influence of codon bias has become weaker with evolution.

## Conclusion

Each RP is a part of a huge RNP complex. Until recently, RP genes were suggested to have a unified control mechanism for transcription and translation. In this study, human RP genes show the following heterogeneity: (1) RP genes show a divided cluster for their gene expression level and some RP genes show tissue-specificity; (2) each RP gene is controlled by different regulators; (3) the optimal amino acids are different in some RP genes; (4) the optimal codon are different in some RP genes. These results demonstrate that RPs have individual characteristics. It can be suggested that certain RP genes have the potential to carry out extra-ribosomal functions as independent polypeptides.

This study to the best of our knowledge is the first attempt to investigate the overall trends in human RP genes. We anticipate elucidating the detailed functions of the RP genes in the future.

## Methods

### Materials

We obtained human and mouse full-length cDNA, genomic DNA, and encoded amino acid sequences from the RPG database [3,4]. Because human RPS4 is encoded on both the X and Y chromosomes, we considered them as two individual RP genes, *RPS4X *and *RPS4Y*. We therefore defined the total number of RP genes, including these, as 80. mRNA expression data were obtained from BODYMAP [23,24], and quantified in 30 tissues by introduced amplified fragment length polymorphism (iAFLP) [41]. Human nucleotide and amino acid sequences, except for those of the RP genes, were collected from RefSeq [21,22].

### Analysis of expression profiles

To investigate the expression profiles, we prepared a total of 3281 gene expression data including those for 63 RP genes from BODYMAP. The RP gene primers used to generate the expression data were verified by comparing their sequences with the corresponding full-length cDNA sequences. The expression levels of 3284 genes were analyzed by hierarchical clustering using Cluster 3.0 software [42] and Java TreeView 1.0.12 [20] with centroid linkage. The clustering algorithm applies equal weight to each gene expression data in all tissues. To find differentially expressed RP genes, these data were standardized by Z-transformation and classified by outlier analyses (*P *< 0.05, Grubbs' test).

### Prediction of transcription factor binding sites

We predicted possible transcription factor binding sites using the human/mouse phylogenetic footprinting method. The 5' flanking regions located between -500 and +500 bp of the TSS of 79 RP genes were analyzed. The position of TSS was determined by comparison of the full-length cDNAs and genomic sequences [9]. The human sliding window (50 bp) was moved 10 bp downstream to the same region in the mouse ortholog and the process repeated to calculate individual identities. The identity in the window was given the maximum alignment score by ClustalW [43] in each position of the mouse RP genes. The window conserved between mouse and human (identity > 60%) was targeted for predicting transcription factor binding sites in order to eliminate false positives. We used MatInspector version 2.1 [44]/ TRANSFAC 3.1 [45] with the default parameters to predict known promoters. We applied the values of parameters, which were relaxed criteria, to predict possible transcription binding sites. We searched possible binding sites that had already been reported in several RP genes, including a GC box (NRGGGGCGGGGCNK), a TATA box (STATAAAWRNNNNNN), and binding sites for NRF-2 (ACCGGAAGNS), YY1 (NNNCGGCCATCTTGNCTSNW), and AP-1 (RSTGACTNMNW). We allowed only the 5'-to-3' direction in prediction of the TATA box and both directions for the other sites.

### Analysis of amino acid and synonymous codon composition

We prepared 3000 amino acid sequences randomly selected from RefSeq (excluding RP genes) and 80 amino acid sequences encoded by RP genes (see Additional file 5). Amino acid composition was calculated by adapting relative amino acid usage (RAAU). We performed hierarchical clustering from the score by using Cluster 3.0 software [42] with centroid linkage. The dendrogram was generated by Java TreeView 1.0.12 [20]. In the analysis of synonymous codon composition, the 3000 randomly selected ORFs from RefSeq (excluding RP genes) and 80 nucleotide sequences of the RP genes were prepared for clustering (see Additional file 8). Codon usage data, termed relative adaptiveness (W) by Sharp and Li, was calculated from the relative synonymous codon usage (RSCU) [40].

RSCUij=obsijaaij/k     (1)
 MathType@MTEF@5@5@+=feaafiart1ev1aaatCvAUfKttLearuWrP9MDH5MBPbIqV92AaeXatLxBI9gBaebbnrfifHhDYfgasaacH8akY=wiFfYdH8Gipec8Eeeu0xXdbba9frFj0=OqFfea0dXdd9vqai=hGuQ8kuc9pgc9s8qqaq=dirpe0xb9q8qiLsFr0=vr0=vr0dc8meaabaqaciaacaGaaeqabaqabeGadaaakeaacqqGsbGucqqGtbWucqqGdbWqcqqGvbqvdaWgaaWcbaGaemyAaKMaemOAaOgabeaakiabg2da9maalaaabaGaee4Ba8MaeeOyaiMaee4Cam3aaSbaaSqaaiabdMgaPjabdQgaQbqabaaakeaacqqGHbqycqqGHbqydaWgaaWcbaGaemyAaKMaemOAaOgabeaakiabc+caViabdUgaRbaacaWLjaGaaCzcamaabmaabaGaeGymaedacaGLOaGaayzkaaaaaa@47D3@

Wij=RSCUij/RSCUimax⁡     (2)
 MathType@MTEF@5@5@+=feaafiart1ev1aaatCvAUfKttLearuWrP9MDH5MBPbIqV92AaeXatLxBI9gBaebbnrfifHhDYfgasaacH8akY=wiFfYdH8Gipec8Eeeu0xXdbba9frFj0=OqFfea0dXdd9vqai=hGuQ8kuc9pgc9s8qqaq=dirpe0xb9q8qiLsFr0=vr0=vr0dc8meaabaqaciaacaGaaeqabaqabeGadaaakeaacqqGxbWvdaWgaaWcbaGaemyAaKMaemOAaOgabeaakiabg2da9iabbkfasjabbofatjabboeadjabbwfavnaaBaaaleaacqWGPbqAcqWGQbGAaeqaaOGaei4la8IaeeOuaiLaee4uamLaee4qamKaeeyvau1aaSbaaSqaaiabdMgaPjGbc2gaTjabcggaHjabcIha4bqabaGccaWLjaGaaCzcamaabmaabaGaeGOmaidacaGLOaGaayzkaaaaaa@484B@

In the above formula, RSCU_*ij *_is the relative synonymous codon usage of codon *j *in sequence *i*. obs_*ij *_is the actual observed number of codon *j *in sequence *i*. aa_*ij *_is the total number of amino acids coded by codon *j *in sequence *i*, and *k *is the number of synonymous codons of codon *j*.

## Authors' contributions

**KI **performed bioinformatic analysis and wrote the manuscripts. **TW **and **NK **initiated the study and revised the draft critically for intellectual content. **TU **and **MY **prepared the dataset, gene expression data and sequences of the RP gene for this study and helped with the writing of the manuscript. **MT **participated on the design and coordination of the study. All authors read and approved the final manuscripts.

## Supplementary Material

Additional File 1 Projection of synonymous codon frequency vectors of ribosomal protein (RP) genes in six species onto the factorial plane formed by the first two principal components. The *X *axis represents the first principal component score and the *Y *axis represents the second principal component score. The numbers of RP genes used in this analysis were as follows. *Homo sapiens *large subunit genes (L): 47, small subunit genes (S): 33; *Drosophila melanogaster *L:57, S:40; *Caenorhabditis elegans *L:50, S:31; *Saccharomyces cerevisiae *L:81, S:56; *Methanococcus jannaschii *L:37, S:25; *Escherichia coli *L:33, S:21. Click here for file

Additional File 2 The data files for the cluster analyses in Gene expression, Amino acid composition and Codon composition. The original data file (gene_expression.txt, amino_acid.txt, codon.txt) prepared for Cluster 3.0. Click here for file

Additional File 3 The data files for the cluster analyses in Gene expression, Amino acid composition and Codon composition. The output file (gene_expression.cdt, amino_acid.cdt, codon.cdt) of Cluster 3.0. To make Figure 1, 4 and 5, these data file were used by the Treeview 1.0.12. The order of the gene names in these files was arranged based on the result of cluster analysis. Click here for file

Additional File 4 The data files for the cluster analyses in Gene expression, Amino acid composition and Codon composition. The output file (gene_expression.gtr, amino_acid.gtr, codon.gtr) of Cluster 3.0. To make Figure 1, 4 and 5, these data file were used by the Treeview 1.0.12. Click here for file

Additional File 5 The data files for the cluster analyses in Gene expression, Amino acid composition and Codon composition. The original data file (gene_expression.txt, amino_acid.txt, codon.txt) prepared for Cluster 3.0. Click here for file

Additional File 6 The data files for the cluster analyses in Gene expression, Amino acid composition and Codon composition. The output file (gene_expression.cdt, amino_acid.cdt, codon.cdt) of Cluster 3.0. To make Figure 1, 4 and 5, these data file were used by the Treeview 1.0.12. The order of the gene names in these files was arranged based on the result of cluster analysis. Click here for file

Additional File 7 The data files for the cluster analyses in Gene expression, Amino acid composition and Codon composition. The output file (gene_expression.gtr, amino_acid.gtr, codon.gtr) of Cluster 3.0. To make Figure 1, 4 and 5, these data file were used by the Treeview 1.0.12. Click here for file

Additional File 8 The data files for the cluster analyses in Gene expression, Amino acid composition and Codon composition. The original data file (gene_expression.txt, amino_acid.txt, codon.txt) prepared for Cluster 3.0. Click here for file

Additional File 9 The data files for the cluster analyses in Gene expression, Amino acid composition and Codon composition. The output file (gene_expression.cdt, amino_acid.cdt, codon.cdt) of Cluster 3.0. To make Figure 1, 4 and 5, these data file were used by the Treeview 1.0.12. The order of the gene names in these files was arranged based on the result of cluster analysis. Click here for file

Additional File 10 The data files for the cluster analyses in Gene expression, Amino acid composition and Codon composition. The output file (gene_expression.gtr, amino_acid.gtr, codon.gtr) of Cluster 3.0. To make Figure 1, 4 and 5, these data file were used by the Treeview 1.0.12. Click here for file
